# Comparing patterns of volatile organic compounds exhaled in breath after consumption of two infant formulae with a different lipid structure: a randomized trial

**DOI:** 10.1038/s41598-018-37210-5

**Published:** 2019-01-24

**Authors:** A. Smolinska, A. Baranska, J. W. Dallinga, R. P. Mensink, S. Baumgartner, B. J. M. van de Heijning, F. J. van Schooten

**Affiliations:** 1 NUTRIM School of Nutrition and Translational Research in Metabolism, Department Pharmacology & Toxicology, Maastricht University, Maastricht, The Netherlands; 2 NUTRIM School of Nutrition and Translational Research in Metabolism, Department of Human Biology, Maastricht University, Maastricht, The Netherlands; 3 Nutricia Research, Utrecht, The Netherlands

## Abstract

Infant formulae have been used since decades as an alternative to or a complement to human milk. Human milk, the “gold standard” of infant nutrition, has been studied for its properties in order to create infant formulae that bring similar benefits to the infant. One of the characteristics of milk is the size of the lipid droplets which is known to affect the digestion, gastric emptying and triglyceride metabolism. In the current study a concept infant milk formula with large, phospholipid coating of lipid droplets (mode diameter 3–5 μm; NUTURIS, further described as “active”), was compared to a commercially available formula milk characterised by smaller lipid droplets, further described as “control” (both products derived from Nutricia). We investigated whether we could find an effect of lipid droplet size on volatile compounds in exhaled air upon ingestion of either product. For that purpose, exhaled breath was collected from a group of 29 healthy, non-smoking adult males before ingestion of a study product (baseline measurements, T0) and at the following time points after the test meal: 30, 60, 120, 180 and 240 min. Volatile organic compounds (VOCs) in breath were detected by gas chromatography-*time-of-flight*-mass spectrometry. Any differences in the time course of VOCs patterns upon intake of active and control products were investigated by regularised multivariate analysis of variance (rMANOVA). The rMANOVA analysis revealed statistically significant differences in the exhaled breath composition 240 min after ingestion of the active formula compared to control product (p-value < 0.0001), but did not show significant changes between active and control product at any earlier time points. A set of eight VOCs in exhaled breath had the highest contribution to the difference found at 240 minutes between the two formulas. A set of ten VOCs was different between baseline and the two formulae at T240 with p-value < 0.0001. To our knowledge this is the first study that shows the ability of VOCs in exhaled breath to monitor metabolic effects after ingestion of infant formulae with different lipid structure. The statistically significant differences in compound abundance found between active and control formula milk may be related to: (i) specific differences in the digestion, (ii) absorption of lipids and proteins and (iii) assimilation of the products in the gut.

## Introduction

Human milk from a healthy mother is considered the most beneficial form of infant nutrition providing the best nutritional components, the most efficient delivery of nutrients and best transfer of immunological protection from a mother to her child. The aim of various producers of infant formula is to improve their current product concept by mimicking, as close as possible, native beneficial characteristics of human milk. The composition of human milk is very complex and dynamic, and fluctuates over time. The content of human milk is associated with the maternal diet and phenotype^1,2^. Physiologically, fresh human milk contains fat globules that are on average about 2–10 times larger than those present in processed regular infant milk formulae. Moreover, the small fat globules present in infant formulae do not contain a phospholipid membrane that normally surrounds fat globules in human milk. In a study by Prentice *et al.*^3^, differences in dried blood spots lipid profiles were shown between human-fed versus formula-fed babies. Similarly, differences in *Bifidobacteria* count in fecal samples were found between formula-fed and human milk-fed infants^4^. Fat globule size influences the digestibility including gastric emptying and triglyceride metabolism^5,6^. Large fat globules are faster released from the stomach and thus result in earlier appearance and higher levels of triglycerides in blood^6^. In rats, this leads to a shift towards utilization of lipids in the metabolism of triglycerides for energy generation instead of incorporation and storage^6–8^. Small fat globules normally found in infant formulae remain in the stomach for a longer time and take more time to be absorbed in the gut. Therefore, they are less likely to be used for energy generation and might be used for fat storage to a large extent^7^. Early postnatal life is regarded as a critical period for the development of the metabolic system, which is programmed in early life in its functionality later in life. The relationship between infant feeding mode and long-term metabolic health could be related to the early-life growth trajectory. More specifically, rapid weight gain in early life has been related to higher risk of visceral adipose tissue in young children and adults^9,10^. A concept infant formula was developed with large, phospholipid coated lipid droplets (Nuturis)^11,12^. The beneficial effect of Nuturis infant formula was shown in a long-term experiment in mice^13^. Oosting and colleagues^13^ showed that exposure to Nuturis early in life, helped mice to cope better with a moderate western style diet later in life, implicating a metabolic imprinting effect of Nuturis. The study demonstrated that the early exposure to the large, phospholipid coated lipid droplet concept diminished the accumulation of adipose tissue and improved the metabolic profile in mice during adolescence.

In the present study, the acute effect of Nuturis is examined in healthy male volunteers. The metabolic response upon ingestion of Nuturis (active) or standard infant milk formula (control) was tested and monitored in an exploratory, randomised, double-blind, controlled, crossover study up to 4 h post-ingestion as reported earlier by Baumgartner *et al*.^14^. Post-meal samples of exhaled air (0–4 h) were collected and analysed for volatile organic compounds (VOCs) as a read-out for metabolic responses. The analysis of the broad range of VOCs in exhaled breath gained popularity over the last years as a diagnostic and monitoring tool^15,16^. Various metabolic processes generate VOCs that are first released into the blood and then excreted via the lungs due to low solubility. In addition, the microbiome present in the gastrointestinal tract generates a number of volatile compounds during the metabolism of nutrients and xenobiotics^15^. Some of these compounds are excreted into faeces, while others enter the systemic circulation where they might be further modified by host metabolism and eventually excreted in breath. A single breath contains 300–500 VOCs occurring in nmol/l-pmol/l concentrations and can be detected with several analytical techniques, including gas chromatography and mass spectrometry based methods^17^. The number of VOCs in a series of breath samples may reach up to 3000–5000 due to inter-individual differences. Recent studies show that VOC profiles in exhaled air change due to, for instance, a gluten-free diet or the amount of fiber in the diet^18,19^. Therefore, we hypothesize that specific VOCs should be detected in exhaled breath after consuming two different formulas milk. In this study, we aim to explore the feasibility of exhaled air samples comparing the postprandial kinetics of two infant formulae, differing only in fat droplet size and phospholipid coating.

## Materials and Methods

### Study participants

As reported previously^14^ 29 healthy, non-smoking adult male with lactose and milk tolerance (age 18–25, BMI 20–25 kg/m^2^) with no current and former known diseases and malfunctions (such as fat malabsorption, gastrointestinal malformation, haemophilia, hepatitis B, HIV, high blood-pressure, hyperlipidemia, diabetes, flue, or severe diarrhoea) and with no current use of any medications nor follow any specific diet participated in the study. The sample size of 29 was termined for a two side effect (p < 0.05) of 20% and a discriminative power of 80%.

All study participants gave written informed consent prior to inclusion. The study protocol was approved by the Maastricht University Medical Center + (MUMC + ) Ethics Committee (13–3–056.5/ab) and was in compliance with the revised Declaration of Helsinki (64th WMA General Assembly, Fortaleza, Brazil, 2013). The trial is registered at the Dutch Trial Registration, http://www.trialregister.nl (clinical trial number: NTR4463, registered on 10.03.2014)^14^.

### Study design

**Figure 1:**
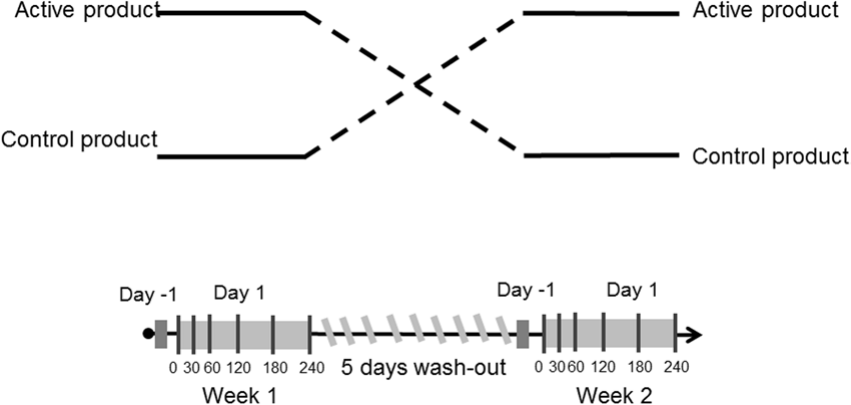
The schematic diagram of the study design.

The study design was randomised, cross-over, and double-blind with nallocation ratio 1:1, and performed in one center at Maastricht University, the Netherlands. The participants were assigned a random number at study entry after which the correspondingly numbered envelope was opend with the sequence of the study product the subject was receiving. The Excel was used for simple randomization of the study product for the participants. Note that the randomisation was unknown to the investigator performing the study and to the site staff. The sequence was, however, known to the statistician who was responsible for making the randomisation sequence, and the Supplies Manager who had to label the study products. The schematic diagram of the study is shown in Fig. 1. The study consisted of two similar test days (day 1 at week 1 and day 1 at week 2). Each test started with a pre-test period (day −1 at week 1 and week 2) of one day preceding the actual study. At day −1 participants were not allowed to sport, drink alcohol or eat high fat meals (i.e. >10 g fat/meal). At the first day of the study (day 1 at week 1) fasting participants were asked to drink prepared shakes (4 times 100 mL within 1 min each and with 1 min interval). The subjects received Nuturis (called active) or control product in randomly assigned order. Both study products were infant milk formula (IMF) and contained an equal fat load of 37.5 g in 400 mL. The active product contained large phospholipid coating of lipid droplets^11,12^, while the control product was commercially available IMF from Nutricia. The breath was collected before ingestion of a study product (baseline measurements, T = 0) and at the following time points after the test meal: 30, 60, 120, 180 and 240 min. After the first day of the study a wash-out period of five days was considered sufficient to erase the effect of one test to another and to start the second analogous test day of the study.

### Exhaled air sampling and analysis

At five time points after intake of the test meal (Fig. 1), and one baseline time point before ingestion, exhaled air was collected from subjects^19^. Subjects exhaled breath into a 3 L Tedlar bag (Tedlar bag, SKC Ltd, Dorset, UK) whose content was thereupon transferred to a carbon-filled stainless steel sorption tubes (Markes International, Llantrisant Business Park, UK) where the breath compounds were trapped and stored until the actual analytical analysis (between 2–6 weeks at room temperature). The content of the sorption tubes was analytically analysed by Gas Chromatography coupled with *time of flight*-Mass Spectrometry (GC-*tof*-MS). The carbon tubes were placed inside the thermal desorption unit (Marks Unity desorption unit, Marks International Limited, Llantrisant, Wales, UK) and quickly heated to 270 °C in order to release all volatile molecules and to transport the released volatiles onto the GC. The volatile compounds trapped on the sorption tubes were released under a flow of Helium using automated thermal desorption. Next, the vaporous mixture was divided into two parts, 90% of the sample was recollected on an identical sample tube and 10% of the sample was transferred to an electrically-cooled sorbent trap (5 °C) from which it was injected into a GC (column: RTX-5ms, 30 m × 0.25 mm 5% diphenyl, 95% dimethylsiloxane, film thickness 1 mm, Thermo Electron Trace GC Ultra, Thermo Electron Corporation, Waltham, USA). The temperature of the GC was programmed as follows: first 40 °C for 5 minutes then rose by 10 °C every minute until 270 °C was reached. This temperature was maintained for 5 minutes. Within the *tof*-MS part electron ionization at 70 eV was applied with a 5 Hz scanning rate over a range of m/z 35–350. The *tof*-MS allows for compounds detection and identification based on their total mass spectrum. *Tof*-MS measures the time necessary to travel in a field free region from the ionization source and acceleration field to a detector plate. Because all ions have the same kinetic energy (E = ½mv^2^), ions with different mass-to-charge ratio (m/z values) are separated in the flight tube based on their velocity. Note, that due to practical limitations, i.e. absence of standard reference materials for a priori unknown VOC, compounds are quantified using relative quantification, i.e. determining the response ratio between a VOC and other VOCs. The output of the GC-*tof*-MS consists of two dimensions: retention time and m/z value and thus, for each measured compound in a breath sample, retention time and mass spectrum are available. These two parameters are also used in the chemical identifications of the VOCs that are selected after multivariate statistical analysis and are considered to contain valuable information^3^. Spectrum recognition using the National Institute of Standards and Technology library in combination with spectrum interpretation by an experienced mass-spectrometrist and identification based on retention times of compounds were used to obtain a putative chemical identification of the relevant VOCs.

### Data pre-processing and statistical analysis

Data pre-processing of the raw data is implemented before the actual statistical analysis, as previously described^20^. Shortly, first the beginning and end of each chromatogram (retention time either < 1.3 or > 23 min) were removed because of noisy mass spectra at the beginning of the chromatograms and column bleeding at the end of each run. Then noise removal was performed via wavelets transformation and baseline correction by means of P-splines. Next step of data pre-processing is the creation of the peak list for each Total ion current (TIC) chromatogram allowing calculation of area under the peaks. Note that these areas are proportional to the relative amounts of measured compounds in exhaled air. Next, the area under the peaks for each TIC chromatograms were merged by combining the corresponding compounds based on retention time and similarities in mass spectra. To make the chromatograms comparable the final step of pre-processing involved PQN normalization^21^.

The postprandial time course of VOCs in expired air after ingestion of the study products were compared and tested for differences. All statistical analyses were performed using Matlab R2014b.

Since each individual delivered 12 breath samples, intra-individual variation may obscure the information of interest. To diminish the influence of intra-individual variation centering per individual was performed. This allows converting all the relative compounds concentrations to fluctuations around zero for each individual instead of around the population mean. In other words, the biological mean for each individual is set to zero^22^.

Before the actual statistical analysis, the presence of outliers is investigated by means of robust-Principal Component Analysis (r-PCA)^23^. The VOCs that are statistically different between active and control product over time and/or at a certain time point were found by means of regularized Multivariate Analysis of Variance (rMANOVA) for paired samples^24^. The rMANOVA technique is an extension of standard MANOVA applicable with poor ratio between number of samples (n) and number of measured parameters (p), here volatiles in exhaled breath.

To visualize the differences, groupings and trends between active and control nutrition product principal component analysis (PCA) was performed on the set of selected significant VOCs. PCA converts the multidimensional data space into a low-dimensional model plane using principal components (PCs). PCs are linear combinations of the originally measured compounds and can be summarized in score plots and loading plots. Score plot provides the summary and the relation of all measured samples in the space defined by PCs. In a loading plot the relation between measured compounds is displayed. The significance of the compounds was tested by means of the Wilcoxon Signed Rank Test^25^ for paired samples, with Benjamini-Hochberg correction^26^. A significance level of p-value < 0.05 was selected.

## Results

### Data

29 individuals delivered exhaled breath samples at five time points and at baseline leading to total number of 348 samples. The initial quality check of the data and r-PCA led to the removal of 35 samples. Therefore, from the total number of 348 samples, the statistical analysis was performed on 313 samples. In these 313 samples a total of 875 compounds was measured. Because the majority of individual VOCs was detected only in a limited number of samples the rule was applied that a compound was only kept for further analysis if it was present in at least 20% of the samples in one of the experimental groups or time points^19^. This led to a final data matrix containing 348 VOCs.

### Active vs. control products

**Figure 2:**
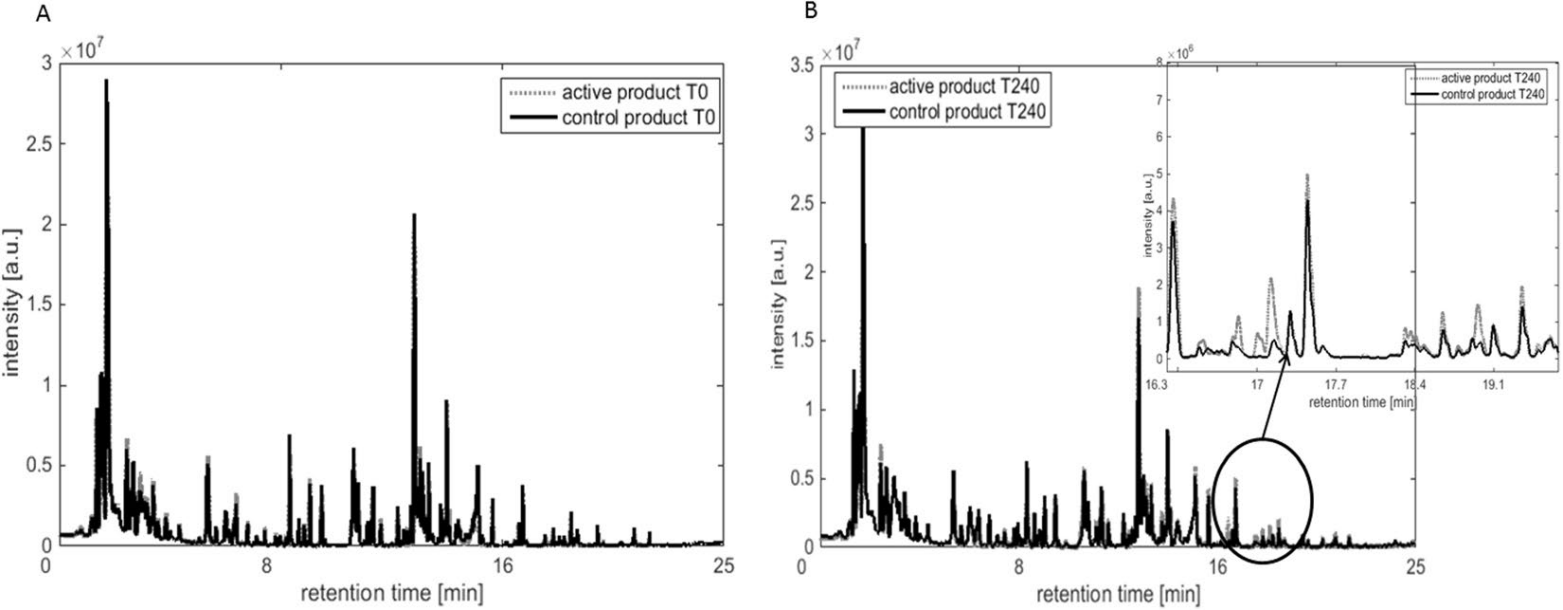
The averaged VOCs profiles for active and control products at: (**A**) baseline T0, i.e. before consumption of the nutrition product; the VOCs profiles of active and control product are similar at the baseline. (**B**) at T240, after consumption of the nutrition product; the VOCs profiles are different between the two nutritional products.

The rMANOVA models were performed on samples obtained at each corresponding time point for the two products (active and control). This analysis showed a significant difference between active and control product only at time point 240 (p-value < 0.0001). None of the earlier time points revealed significant differences in VOCs profiles between the two formulae. In Fig. 2A,B the averaged VOCs profiles for active and control products at baseline T0 and T240 are shown, respectively. As could be expected the VOCs profiles of both groups are similar (Fig. 2A) at baseline, i.e. before ingestion of the formula. Four hours (240 minutes) after consuming the nutrition product the averaged VOCs profile revealed visible differences, as shown in Fig. 2B. The largest differences can be seen in the chromatograms between RT of 16 and 19 min.

**Figure 3:**
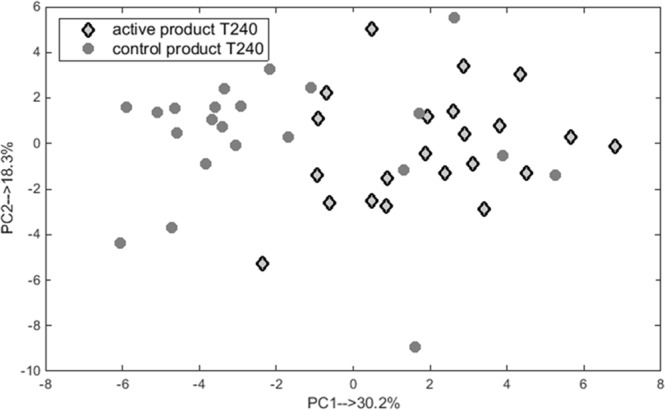
PCA score plot obtained using eight significant VOCs excreted in breath samples taken at T240 after consumption of active and control products. The samples are marked with respect to the nutritional product (diamonds for active and circles for control products).

A set of eight VOCs was selected to be statistically significant (p-value < 0.0001) between active and control products by rMANOVA at time point 240. This set was re-analysed in a separate PCA analysis and the corresponding PCA score plot is shown in Fig. 3. Each diamond and circle point corresponds to a breath sample of an individual consuming an active or control nutritional product. The PCA score plot shows differences between the two products along PC1 and PC2 explaining 48% of the total variance. Six samples from the control nutritional group seems to have a profile of compounds similar as those from the active group as they can be found in the cluster of samples of the active product group. The changes in the relative concentration of each of eight significant compound is demonstrated in Table 1. Three compounds out of eight (2-methyl-propanoic acid, 2,4-dimethylhexane and C_5_H_7_COOH) point in the direction of active product, indicating higher concentrations of these breath compounds in the active group. The opposite is seen for the remaining compounds as shown in Table 1.

**Table 1:** List of 8 VOCs which changes in concentrations were significantly different between both products at T240. (−) indicates decrease in VOC concentration, while (+) indicates increase in active product compared to the control group.

Nr.	Putative identification	Relative change	p-value
1	Isoprene	(−)	0,006
2	C_8_H_18_ branched^#^	(−)	0,006
3	2-methyl-propanoic acid	(+)	0,007
4	2,4-dimethylhexane	(+)	0,006
5	2- or 3-methylthiophene^#^	(−)	0,02
6	Decalactone	(−)	0,006
7	C_5_H_7_COOH^#^	(+)	0,007
8	1,2,4-trimethylbenzene	(−)	0,02

P-value indicates the statistical significance of the compounds.

^#^The exact isomer could not be determined.

Table 1 shows the identified and unidentified VOCs and their relative concentrations change in breath samples obtained from individuals on active versus control product at T240. Up or down regulation of VOC concentrations is indicated as (+) or (−), respectively, with reference to control nutrition product at T240.

Though it is the combination of all 8 VOCs that yields high differences, we also analyzed the statistical importance of the 8 individual VOCs between both products using the Wilcoxon Signed Rank Test. Interestingly, all 8 compounds were statistically significant different, with a p-value ≤ 0.05 (Table 1).

The analysis by rMANOVA did not reveal significant differences between both products at earlier time points (i.e. T0, T30, T60, T120 and T180). The differences observed at T180 were on the border of being significant with a p-value of 0.054. The PCA analysis performed on the set of 8 significant volatiles at T180 did not reveal clear clustering of the groups.

### T0 vs. T240 for active and control product

**Figure 4:**
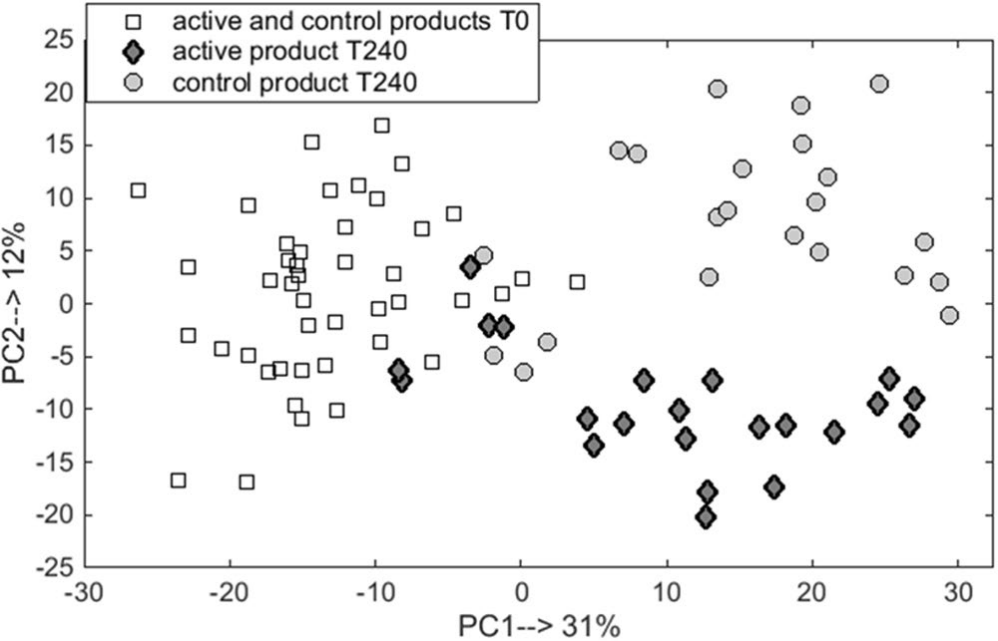
PCA scores plot for breath samples measured at baseline T0 and T240 for active and control products based on 16 volatile compounds. The samples are coded with respect to time (square for T0) and product (diamonds for active and circles for control at T240).

In a second approach, the rMANOVA analysis selected a set of 10 VOCs (p-value < 0.0001) that differ significantly between exhaled breath taken at baseline T0 and T240 (both nutrition products taken together). Two of these compounds were also part of the initial set of 8 VOCs that differentiated between active and control products at T240. Both sets of volatiles were combined, leading to a new set of 16 VOCs and PCA analysis of all T0 and T240 samples was performed on this combined set. The resulting PCA score plot is shown in Fig. 4. The variance observed along PC1 (explaining 31% of total variance) corresponds to differences in VOC profiles between T0 and T240 measurements (both products) while PC2, explaining 12% of variance represents the differences between active and control group at T = 240. For the comparison purpose two additional combinations of PCA score plots were performed, namely: (i) for samples obtained at T240 for active and control product using a set of 16 VOCs, and (ii) for samples obtained at T0 and T240 for active and control product using a set of eight VOCs. The corresponding PCS score plots can be found in the supplementary material (see Figs 1S, 2S). In Figs 1S and 2S, the active and control products create the distinct cluster of samples, indicating differences in volatile metabolites profile.

**Figure 5:**
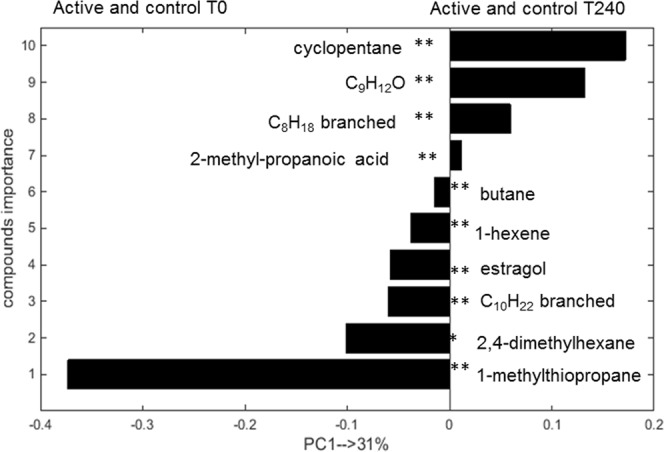
PCA loading bar plot depicting the relative importance of the 10 significant volatiles in the separation of all T0 and T240 breath samples for both products. Bar length is proportional to compound importance. Bars projecting to the left indicate greater abundance in breath samples taken at T0, while those projecting to the right indicate greater abundance in breath samples taken at T240. **p-value < 0.0001, *p-value < 0.05. The exact isomers of C_9_H_12_O, C_10_H_22_ and C_8_H_18_ could not be determined.

As indicated before, a set of 10 VOCs was found to be statistically significant by rMANOVA and these VOCs contribute to the differentiation between baseline measurements and measurements at T240 (i.e. fasting versus postprandial) of both product groups. The relative importance and putative identification of each of these 10 volatile compounds is presented in Fig. 5. Four of these volatiles were more abundant in breath of individuals after consuming one of the products at T240 relative to the baseline measurements. The significance of the individual volatiles is shown in Fig. 5. Nine out of 10 compounds were statistically significant with p-value < 0.0001, while one compound had a p-value < 0.05 (Fig. 5).

From the first set of 8 VOCs 7 were putatively chemically identified, while for the one remaing compound the exact isomer remained unknown. From the set of 10 VOCs, 8 were chemically identified, whereas for the remaining two the exact chemical formula remained unknown. Some compounds could not be chemically identified due to insufficient mass spectrum, overlap in the retention time or absence of mass spectrum in the library.

## Discussion

In this study we demonstrate changes of exhaled VOCs in breath after consumption of different infant formulae. This finding opens possibilities of alternative, non-invasive ways of investigating and monitoring postprandial effects of nutritional products, for instance, in infants and small children. We studied the effect of two infant milk formulae, active (Nuturis containing large phospholipid coating of lipid droplets) and control (commercially available IMF from Nutricia) product, up to 4 hours upon ingestion, in a group of healthy, non-smoking males on the postprandial profile of VOCs in exhaled air. We found that a limited number of eight VOCs to be statistically different between the active and control nutritional products at T240 only, not at earlier time points. In an adult person, the transit time for food to pass from oral intake and subsequent fecal excretion varies between 24–72 hours. In this course, the gastric emptying takes about 2–6 hours and food enters the small intestine where it takes about 3 hours to empty half of its content. Our results show that the largest difference in breath VOC composition between the two formulae was observed after 4 hours (p-value < 0.0001), i.e., the time that food enters the small intestine and nutrients might be absorbed there. Marginal significance was shown at earlier time point, i.e. T180 (p-value = 0.054). Although, the results showed statistical differences after 4 hours of consuming the test products, it is unknown till when the differences hold, since no further time points were sampled. Moreover, the differences shown here are demonstrated for healthy males and therefore it is not completely sure what the outcomes will be if infants are investigated.

The main study outcome reported by Baumgartner *et al*.^14^ shows that consuming active product results in an earlier peak of plasma glucose, insulin levels and an earlier time to nadir in non-esterified fatty acids plasma concentrations In addition, plasma triacylglycerol levels were slightly significantly elevated after 3 hours. The differences in these parameters and VOCs excretion after 3 to 4 hours might be explained by dissimilarities in absorption due to different fat globules and the phospholipid coating. Another finding of our study was that, a set of ten VOCs in exhaled air was found to be statistically significant after 4 hours (T240) compared with before consumption. They might well reflect differences between the fasting stage and the satiation/digestion stage. In addition, we tested whether there are significantly different compounds between T0 and T240, separately, for active and control product (data not shown). In the set of most significant VOCs we found two compounds, 2-methyl-propanoic acid and C_5_H_7_COOH, which were significantly different between active and control product at T240 as well contributing to the differentiation between T0 and T240 when active and control products were combined as one group. This indicates that those compounds might be specific to consuming of the active product.

We found the levels of cyclohexane and estragol to be significantly different between T0 and T240 measurements. Moreover, the levels of decalactone and 1,2,4-trimethylbenzene were significantly lower after consumption of active compared to the control product. The origin of these four compounds is not entirely certain but heterocyclic and aromatic compounds might be of dietary origin (mostly from plants) or air pollution^27^. The fat content of both active and control formulae consist of a blend of vegetable oils indicating that these heterocyclic and aromatic compounds are presumably originating from diet.

One of the selected compounds is 2-methylpropanoic acid (isobutyric acid), which was found to be statistically significantly higher in the active than in the control group at T240, and also significantly higher at T240 (combined groups) compared to T0 measurements. This compound is a structural isomer of butyric acid, which is a short chain fatty acids produced by colonic bacteria during fermentation processes of undigested carbohydrates^28,29^. 2-methylpropanoic acid belongs to the group of branched short chain fatty acids (BSCFAs), which might arise from dissimilation of amino acids^30^. Similarly, the levels of C_5_H_7_COOH (unsaturated fatty acid, for which the exact isomer could not be determined) was found to be significantly elevated in the active group compared to the control group. Although the formula milk is known to contain saturated and unsaturated fatty acids, the source of this compound in exhaled breath remains elusive.

The amount of 1-methylthiopropane was found to be lower after consumption of active or control product at T240 when compared to baseline measurements. 1-methylthio-propane passes into alveolar air from the blood, where it may originate from drug or diet^31^. However, in the current study the healthy volunteers did not take any drugs, and a plausible explanation of the sulfur compounds’ source cannot be offered. Another sulfur-containing compound, 2(or 3)-methylthiophene, was found to be significantly lower in the active compared to the control group. Various sulfur-containing compounds were previously reported in breath, faeces, blood and urine^32–34^ and their origins were linked to sulphate-reducing bacteria, Clostridia in the gut, vegetables, nut, bread and beer in the diet.

A large number of linear and branched hydrocarbons have been found in breath of healthy as well diseased individuals^16,33–35^. Unsaturated fatty acids consumed in our diet can be expected to create many hydrocarbons by chain cleavage and peroxidation^33^. Various hydrocarbons are stable end products of lipid peroxidation and have low solubility in blood, so within a few minutes upon their generation they will be excreted via breath. We found several branched and linear hydrocarbons to be different between T0 and T240 measurements as well as between the two study products. For instance, butane level was found to be reduced in active and control product at T240 in comparison to baseline measurements. Although, butane has been related to inflammatory processes occurring in the body^36^, it is also known that it is produced by the gut microbiome^16^.

Isoprene is one of the most abundant endogenous compounds found in exhaled air^37^. Isoprene is a by-product of cholesterol biosynthesis along the mevalonic acid pathway^33,38^. The level of isoprene in exhaled breath is proportional to the level of cholesterol in blood and therefore there has been a lot of investigation towards the use of breath isoprene analyses as a non-invasive diagnostic tool to measure blood cholesterol levels or cholesterol synthesis rate^39^. The link between isoprene and cholesterol metabolism is further sustained by the observation that there is an instantaneous decrease in isoprene excretion and sterol synthesis upon the administration of lovastatin, a lipid-lowering drug. Therefore, a diet which is rich in cholesterol is responsible for a reduction of isoprene in exhaled breath^40^, hence monitoring the isoprene level in exhaled breath might be a monitoring marker of lipid and cholesterol status^16^. The level of isoprene was found to be significantly lower after the consumption of Nuturis (active product) in comparison to control product at T240. This result is particularly interesting since this would indicate that the sterol synthesis is lower in the active group in comparison to control. As the active meal contained 50% more cholesterol than the control meal, the endogenous cholesterol synthesis might be more inhibited, hence leading to lower isoprene co-production levels. Moreover, active product (Nuturis) contains larger sized fat droplets than control, causing its passage from the stomach to the duodenum to be faster, leading to an accelerated uptake of fat into the blood. This may suggest that after consuming the active product the plasma levels of lipid and cholesterol rise faster in comparison to the control product. This finding is in line with Baumgartner *et al*.^14^ who reported faster increase in blood postprandial triacylglycerol after intake of active product in the same group of participants.

The use of GC-MS in the future implementation of the presented study in infants might be visible but selecting another approach might be more optimal. GC-MS methodology is an offline approach where content of exhaled breath is pre-concentrated on sorption tube and later analytically measured^41^. An alternative technique, capable of detecting in sensitive and fast manner of various volatile metabolites in exhaled breath, is multi capillary column- ion mobility spectrometry. This methodology seems more applicable in infants since the content of breath can be measured directly and it takes about 5–10 minutes for complete analysis of exhaled air. This method has been successfully applied to various fields such as process control^42,43^, biological analysis^44,45^ and medical application^46,47^. In the current study, the various time points were compared individually to each other. However, another approach could be applied, where a longitudinal aspect of the data is taken into account to look for the relevant compounds in exhaled breath that change over time. Recently, Hauschild *et al*. proposed a novel algorithm to first find the compounds that change over time and to define the slope of their change in exhaled breath^48^.

In summary, we have shown the postprandial effect of two infant formulae (active and control) on the VOC composition of exhaled breath. Some of these VOCs may be indicative of specific differences in the digestion and absorption of the two studied products or the role of the gut microbiome in the assimilation of the products. Both should be subject of further investigations.

## Supplementary information

Figure 1S and Figure 2S
