# Supplementary material for: Comparing patterns of volatile organic compounds exhaled in breath after consumption of two infant formulae with a different lipid structure: a randomized trial
Source: Sci Rep. 2019 Jan 24;9:554. doi: 10.1038/s41598-018-37210-5 (PMC6346115; doi:10.1038/s41598-018-37210-5)
Supplement: Supplementary file 1 — Figure 1S and Figure 2S [file 41598_2018_37210_MOESM1_ESM.docx]

Comparing patterns of volatile organic compounds exhaled in breath after consumption of two infant formulae with a different lipid structure: a randomized trial

A. Smolinska^1*^, A. Baranska^1^, J. W. Dallinga^1^, R.P. Mensink^2^, S. Baumgartner^2^, B.J.M. van de Heijning^3^, F.J. van Schooten^1^

^1^NUTRIM School of Nutrition and Translational Research in Metabolism, Department Pharmacology & Toxicology, Maastricht University, The Netherlands.

^2^NUTRIM School of Nutrition and Translational Research in Metabolism, Department of Human Biology, Maastricht University, The Netherlands.

^3^Nutricia Research, Utrecht, The Netherlands

*Corresponding author:

Dr Agnieszka Smolinska

Department of Pharmacology and Toxicology

Maastricht University

PO Box 616

6200 MD Maastricht, The Netherlands

A.Smolinska@maastrichtuniversity.nl

+31-433882495

Figure 1S. PCA score plot obtained using 16 significant VOCs excreted in breath samples taken at T240 after consumption of active and control products. The samples are marked with respect to the nutritional product (diamonds for active and circles for control products).

Figure 2S. PCA scores plot for breath samples measured at baseline T0 and T240 for active and control products based on eight volatile compounds. The samples are coded with respect to time (square for T0) and investigated products (diamonds for active and circles for control at T240).
